# Enhanced Solid-State Biogas Production from Lignocellulosic Biomass by Organosolv Pretreatment

**DOI:** 10.1155/2014/350414

**Published:** 2014-08-05

**Authors:** Safoora Mirmohamadsadeghi, Keikhosro Karimi, Akram Zamani, Hamid Amiri, Ilona Sárvári Horváth

**Affiliations:** ^1^Department of Chemical Engineering, Isfahan University of Technology, Isfahan 84156-83111, Iran; ^2^Industrial Biotechnology Group, Institute of Biotechnology and Bioengineering, Isfahan University of Technology, Isfahan 84156-83111, Iran; ^3^Swedish Centre for Resource Recovery, University of Borås, 50190 Borås, Sweden

## Abstract

Organosolv pretreatment was used to improve solid-state anaerobic digestion (SSAD) for methane production from three different lignocellulosic substrates (hardwood elm, softwood pine, and agricultural waste rice straw). Pretreatments were conducted at 150 and 180^°^C for 30 and 60 min using 75% ethanol solution as an organic solvent with addition of sulfuric acid as a catalyst. The statistical analyses showed that pretreatment temperature was the significant factor affecting methane production. Optimum temperature was 180^°^C for elmwood while it was 150^°^C for both pinewood and rice straw. Maximum methane production was 152.7, 93.7, and 71.4 liter per kg carbohydrates (CH), which showed up to 32, 73, and 84% enhancement for rice straw, elmwood, and pinewood, respectively, compared to those from the untreated substrates. An inverse relationship between the total methane yield and the lignin content of the substrates was observed. Kinetic analysis of the methane production showed that the process followed a first-order model for all untreated and pretreated lignocelluloses.

## 1. Introduction

Worldwide concerns about the limitations of fossil resources, rising crude oil prices, and greenhouse gas (GHG) emissions have led researchers to seek alternative clean and renewable energy sources, for example, biofuels [1]. Lignocellulosic materials are abundant and renewable feedstocks that have recently been considered for the production of biofuels [2–6]. Compared to liquid biofuels, biogas has been shown to have far better performance with respect to both agricultural land area efficiency and life cycle assessments [7].

Biogas, produced during anaerobic digestion (AD) processes, can be used as a versatile source of energy to produce heat and electricity, either separate or combined, and to propel vehicles. The production of biogas offers other advantages, such as controlling organic waste, reducing greenhouse gas emissions, and producing another economically viable fertilizer [8, 9]. AD processes are classified into liquid anaerobic digestion (LAD) and solid-state anaerobic digestion (SSAD), based on the solid content [10]. LAD operates at a total solid (TS) content of less than 15%, while SSAD is generally called for a TS content of higher than 15% [11]. Smaller specific reactor volume, fewer moving parts, lower energy input for heating, easier handling of the end product, and lower parasitic energy loss are the main advantages of SSAD in comparison with LAD [11–13]. SSAD is specially required with lignocellulosic feedstocks, such as agricultural residues with low moisture content [11, 14]. However, the anaerobic digestion of lignocelluloses is limited by the rate of hydrolysis due to their recalcitrant structure [15]. Therefore, an additional pretreatment process is essential to improve their digestibility [15, 16].

Although different factors, for example, the crystallinity of cellulose and the accessible surface area, may play important roles in the bioconversion of lignocelluloses, the presence of lignin is apparently the most important factor affecting biodegradability [17–20]. The lignin-carbohydrate matrix limits the digestibility of lignocelluloses since lignin is a hydrophobic polymer that forms a cross-linked network among the carbohydrates. This network is highly resistant to enzymatic and microbial degradations [6, 21]. Hence, the biogas production from lignocelluloses can be improved by a delignification process. Removal of lignin by ethanol is among the most efficient pretreatment techniques in improving bioconversion of lignocelluloses [22, 23]. Furthermore, since lignin is a value added by-product, an additional unique benefit of organosolv pretreatment is unaltered lignin separation [23]. Therefore, using ethanol as an organosolv for pretreatment prior to the AD process has been reported to improve the economy of the process by increasing methane yield and recovery of lignin [24]. To our knowledge, there is no publication in the literature on utilizing organosolv pretreatment prior to SSAD of lignocelluloses.

The main objective of this study was to improve the performance of solid-state anaerobic digestion of three different types of lignocelluloses, that is, elmwood, pinewood, and rice straw, by applying organosolv pretreatments using ethanol under varying conditions. The effects of the pretreatment parameters, that is, temperature and duration time, on the methane yield were determined by solid-state batch anaerobic digestion assays. In addition, the kinetics of the degradation process was investigated for both untreated and pretreated substrates.

## 2. Material and Methods

### 2.1. Feedstocks and Inoculum

Elm, a hardwood, pine, a softwood, and rice straw, an agricultural waste, were used as substrates for biogas production. Elmwood and pinewood were obtained from the forest of Isfahan University of Technology (Isfahan, Iran), and rice straw (Sazandegi cultivar, Isfahan, Iran) was sourced from a field in Lenjan Province, Iran. Both elmwood and pinewood were debarked, cut into smaller pieces, and milled to obtain chips of less than 2 cm. The wood chips and the rice straw were partly ball-milled and screened to achieve powder with particle sizes between 295 and 833 *μ*m (20–80 mesh). The screened substrates were then stored in airtight plastic bags at room temperature until use.

Effluent of a 7000 m^3^ mesophilic anaerobic digester (Isfahan Municipal Sewage Treatment, Isfahan, Iran) was used as inoculum for the batch digestion assays. Due to its low TS content, the inoculum was centrifuged at 4500 rpm for 30 min to obtain the desirable TS content for the SSAD. The supernatant was discharged, and the remaining sludge was mixed to obtain a homogenous inoculum for SSAD. The inoculum was kept at 37°C for one week for stabilization.

### 2.2. Organosolv Pretreatment

Ethanol as an organic solvent together with sulfuric acid as catalyst was used for the pretreatments. A predetermined amount of each feedstock was mixed with 75% (v/v) aqueous ethanol solution supplemented with 1% w/w (based on dry mass) sulfuric acid to obtain a solid-to-liquid ratio of 1 : 8 (based on dry mass). The pretreatments were carried out in a 500 mL high-pressure stainless steel batch reactor [25]. After loading the substrate and the acidic ethanol mixture, the reactor was heated at a rate of 3°C/min to the desired temperature, that is, 150 or 180°C, and this temperature was held for 30 or 60 min. Then, the reactor was cooled in an ice bath. Afterwards, the pretreated materials were removed, washed three times with 100 mL aqueous ethanol (75% v/v, 60°C), and left overnight to air dry [24, 26]. The pretreated materials were stored in airtight plastic bags at room temperature until use.

### 2.3. Solid-State Anaerobic Digestion (SSAD) and Modeling

The untreated and pretreated elmwood, pinewood, and rice straw (1 g dry mass) were mixed with a predetermined amount of inoculum and deionized water to achieve a feed-to-inoculum ratio (F/I) (based on volatile solids (VS) content) of 3 and initial TS content of 21%. Sealable 118 mL glass reactors were used for the anaerobic digestion assays. Anaerobic conditions were provided by purging the reactors with nitrogen gas for about 2 min, and the reactors were then incubated in a convection oven at mesophilic conditions (39 ± 1°C) for 55 days [27]. Inoculum (without adding any substrate) was evaluated as a blank to determine the inoculum's methane production. All digestion assays were run in duplicate. Gas samples were taken and analyzed for produced biogas volume and composition in every 3 days during the first 9 days of the experimental period and then in every 5 or 6 days until 55 days.

The kinetics of the anaerobic digestion process was also evaluated using a first-order kinetic model (1). The first-order kinetic model was linearized as shown in (2) [28]:

(1) $$\begin{array}{c} -\frac{dM}{dt}=kM, \end{array}$$

(2) $$\begin{array}{c} \ln\left(\frac{M_{u}}{M_{u}-M_{t}}\right)=kt, \end{array}$$

where *t* (day) is time and *M*_*u*_ and *M*_*t*_ (L*·*kg^−1^CH) are methane yields obtained in 55 days and *t* days, respectively, and *k* is the specific rate constant.

### 2.4. Analytical Methods

Total solid (TS) and volatile solid (VS) contents of the feedstocks and inoculum were measured by drying the samples at 105°C followed by heating the dried residues at 575°C to a constant weight [17]. The untreated and pretreated samples were analyzed for lignin and hemicellulose contents according to the methods presented by Sluiter et al. [29] and Yang et al. [30], respectively. The cellulose content was calculated as the remaining TS, based on an extractive-free basis, assuming that ash, hemicellulose, lignin, and cellulose are the only components of the entire biomass.

Methane and carbon dioxide produced during the anaerobic digestions were analyzed by a gas chromatograph (Sp-3420A, TCD detector, Beijing Beifen Ruili Analytical Instrument Co., China) equipped with a packed column (3 m length and 3 mm internal diameter, stainless steel, Porapak Q column, Chrompack, Germany). The carrier gas was nitrogen at a flow rate of 45 mL/min. The column, injector, and detector temperatures were 40, 100, and 150°C, respectively. A pressure-tight syringe (VICI, Precision Sampling, Inc., USA) with a volume of 250 *μ*L was used for gas sampling and injection, enabling taking of gas samples at the bioreactors' actual pressure. Excess gas was released through a needle after each gas sampling to avoid overpressure built-up in the bottles.

All biogas yields were presented at standard conditions.

### 2.5. Statistical Analysis

Analysis of variance (ANOVA) using Minitab software v. 15 was performed to compare confidence intervals and significance between treatments. The factors were considered significant when the probability (*P* value) was less than 0.05.

## 3. Results and Discussion

### 3.1. Characterization of Inoculum

The inoculum obtained from the industrial biogas plant contained 5.7 and 2.7% TS and VS, respectively (Table 1). In order to achieve a TS content of 21% in SSAD, the inoculum was centrifuged [31] to reach TS and VS contents of 11.7% and 5.3%, respectively (Table 1).

### 3.2. The Effect of Different Pretreatment Conditions on the Composition of Substrates

Elmwood, pinewood, and rice straw were subjected to organosolv pretreatment using ethanol prior to anaerobic digestion in order to improve the yield of biogas production. The untreated and pretreated materials were characterized, according to their TS, VS, lignin, cellulose, and hemicellulose contents, and results are summarized in Table 1.

Total lignin contents of untreated elmwood and pinewood were 26.2 and 26.8%, respectively, which was much higher than that of untreated rice straw (17.1%).

The various components of the materials were differently affected by the pretreatments. Depending on the pretreatment conditions, the lignin contents were reduced by 4–27% for elmwood, by 1–21% for pinewood, and by 21–37% for rice straw. Increasing the severity of the pretreatment generally resulted in higher lignin removal. A relatively high portion of straw's lignin (37.7%) was removed through pretreatment at 180°C for 60 min, resulting in a pretreated straw with carbohydrate content of over 77% of TS. On the other hand, the organosolv pretreatment of elmwood and pinewood, at 180°C for 60 min, resulted in 27% and 21% lignin removal, respectively, with corresponding CH contents of 72.7% and 72.5% of TS, respectively. In addition to delignification, parts of hemicelluloses were also removed due to the pretreatments. Higher hemicellulose removal was obtained in pretreated pinewoods (28–40%), compared to that in elmwood (11–19%) or straw (9–16%).

### 3.3. Biogas Production

**Figure 1:**
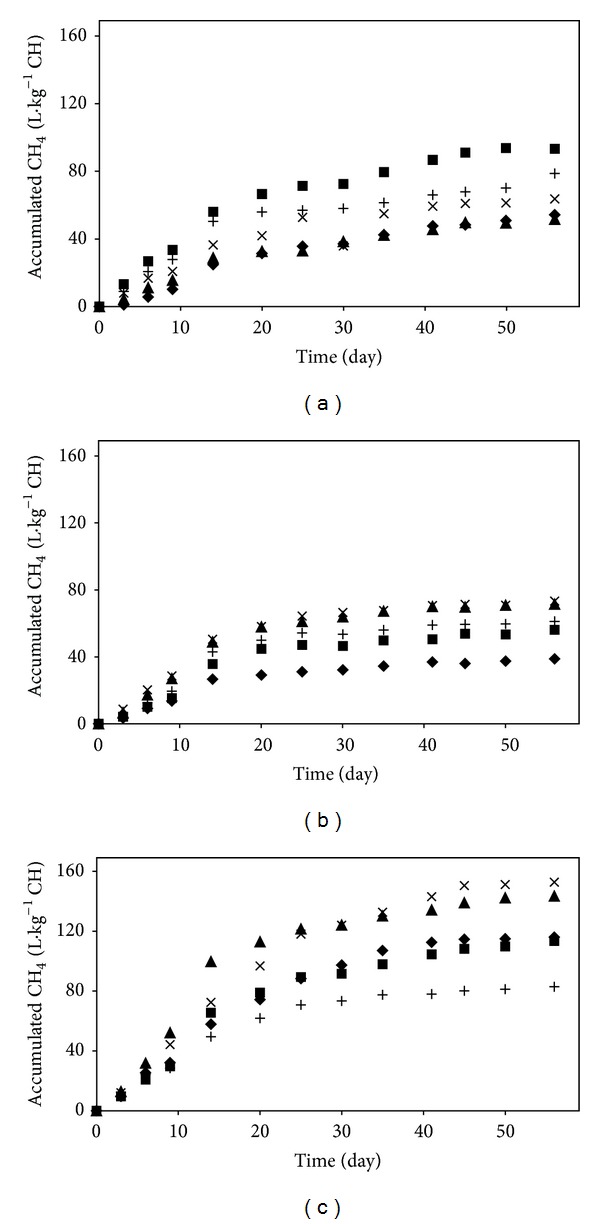
Accumulated methane production from SSAD of untreated and pretreated (a) elmwood, (b) pinewood, and (c) rice straw in different pretreatment conditions. The symbols represent the untreated substrates (◆), the substrates pretreated at 150°C for 0.5 h (▲), at 150°C for 1 h (×), at 180°C for 0.5 h (+), and at 180°C for 1 h (■).

Organosolv pretreatments in four different conditions were performed on the three different lignocellulosic materials, and the methane yields of the pretreated and untreated materials were then measured through batch SSAD assays. The accumulated methane productions obtained during 55 days of digestion from the untreated and pretreated materials are shown in Figure 1.

Methane production yields from all of the substrates were generally improved by the pretreatments in all conditions. The highest methane yield of 152.7 L*·*kg^−1^CH was obtained from rice straw pretreated at 150°C for 1 h (Table 2). However increasing the pretreatment temperature resulted in a reduced methane yield. This could be due to the inhibitory products which can be formed at high temperature during the pretreatment. In contrast, the highest yield of methane production from pretreated elmwood (93.7 L*·*kg^−1^CH) was obtained after pretreatment at 180°C for 1 h; hence, the methane production from elmwood was improved by increasing the severity of the pretreatment. However, the pretreatment of pinewood at 150°C for 0.5 h (the lowest severity) resulted in a methane yield of 71.4 L*·*kg^−1^CH, which showed 84% improvement compared to the methane yield from untreated pinewood. Although pretreating pinewood had a remarkable effect on the yield of methane production (i.e., improvements of 45–84%), the statistical analyses using methane yield as response variable showed that neither temperature nor time, with *P* values of 0.28 and 0.91, respectively, had a significant effect on methane yield in the case of pinewood. In contrast, pretreatment temperature had a significant effect on the methane production from elmwood and rice straw; while being similar to that of pinewood, it was concluded that the effect of pretreatment time on methane production from elmwood, pinewood, and rice straw was not significant (*P* values of 0.14, 0.91, and 0.27, resp.).

Among the untreated samples, the highest methane yield, 115.9 L*·*kg^−1^CH, was obtained from rice straw, which had the lowest lignin content among the substrates utilized in this study. The digestion of untreated elmwood and pinewood resulted in methane yields of 54.2 and 38.7 L*·*kg^−1^CH, respectively. The presence of pores in the structure of hardwoods which facilitate microorganisms' accessibility might be responsible for the higher yield obtained from elmwood in comparison to that from pinewood [32].

### 3.4. Methane Production Modeling

The fitting of kinetics data on the first-order model for all of the substrates is shown in Table 2, as well as the accumulated methane yields obtained after 55 days of SSAD. The regression coefficients demonstrated that methane production followed the first-order kinetic model (*r* ^2^ > 0.91). At the optimum pretreatment conditions for each substrate, that is, 180°C and 1 h, 150°C and 0.5 h, and 150°C and 1 h for elmwood, pinewood, and rice straw, the corresponding *k* value was at its maximum level, respectively, representing the highest degradation rate for each investigated substrate.

### 3.5. Relationship between Total Lignin Content and Methane Yield from Lignocellulosic Materials

**Figure 2:**
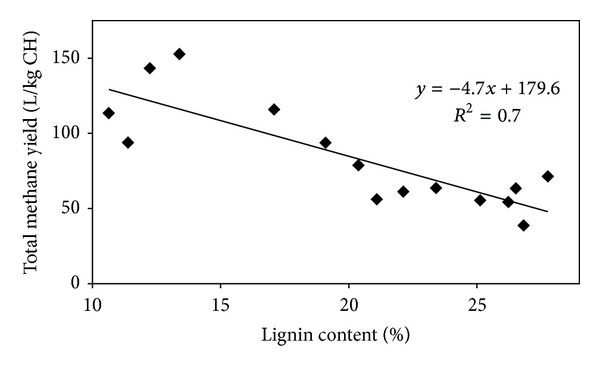
Relationship between lignin content and total methane yield from lignocellulosic substrates (untreated and pretreated elmwood, pinewood, and rice straw).

The effect of lignin content on final methane yield was investigated by comparing methane yield as a function of the materials' lignin content (Figure 2). In line with a previous study [28], an overall inverse relationship between the lignin content of different substrates and the achieved methane yields was observed. However, the low linear regression coefficient of 0.7 confirmed that the content of lignin is not the sole key factor affecting methane yield. The contents of cellulose and hemicellulose, the crystallinity of cellulose, and the accessible surface area may also play important roles affecting methane yields [17–20]. Therefore, further investigations are required to find the specific reason for the observed improvements.

## 4. Conclusions

Organosolv pretreatment prior to SSAD was an efficient process for improvement of methane production from different types of lignocellulosic materials; however, its effectiveness greatly depended on the type of lignocelluloses. The pretreatment process was more effective on softwood than on hardwood or agricultural waste. Moreover, hardwood needed more severe conditions to be able to achieve maximum improvement during the subsequent batch digestion assays. Lignin content was among the most important factors negatively affecting the methane production from all of the investigated lignocellulosic substrates.

## Figures and Tables

**Table 1:** Composition analyses of the inoculum as well as the untreated versus pretreated feedstocks.

Samples	Pretreatment	TS content (%)	VS content (%)	Total lignin∗ (%)	Hemicellulose (%)	Cellulose (%)
Inoculum	—	5.7	2.7	ND	ND	ND
	Centrifuged	11.7	5.3	ND	ND	ND

Elmwood	Untreated	95.5	94.5	26.2	26.3	46.4
	150°C, 0.5 h	95.5	94.1	25.1	23.4	50.0
	150°C, 1 h	95.5	93.8	23.4	21.5	53.3
	180°C, 0.5 h	96.3	94.4	20.4	21.9	55.7
	180°C, 1 h	94.9	93.6	19.1	21.3	58.1

Pinewood	Untreated	95.1	95.2	26.8	28.0	44.5
	150°C, 0.5 h	95.3	94.6	27.8	20.2	51.3
	150°C, 1 h	95.9	95.1	26.5	21.3	51.4
	180°C, 0.5 h	96.5	95.5	22.1	18.5	58.4
	180°C, 1 h	96.9	95.8	21.1	16.9	60.8

Rice straw	Untreated	95.4	83.9	17.1	50.1	21.5
	150°C, 0.5 h	95.6	83.8	12.2	45.6	29.9
	150°C, 1 h	95.7	83.6	13.4	45.3	28.7
	180°C, 0.5 h	95.9	86.2	11.4	42.3	36.2
	180°C, 1 h	96.0	84.7	10.6	42.2	35.3

ND = not determined.

∗Sum of acid soluble lignin (ASL) and acid insoluble lignin (AIL) contents.

**Table 2:** The accumulated methane yields obtained after 55 days of anaerobic digestion from untreated and pretreated lignocellulosic substrates together with the specific rate constants and the regression coefficients calculated from the first-order kinetic model fitting.

Sample	Pretreated conditions	CH_4_ (L*·*kg^−1^CH)	*k* (day^−1^)	*r* ^2^
Elmwood	Untreated	54.2 ± 3.5	0.054	0.975
	150°C, 0.5 h	55.4 ± 9.7	0.063	0.934
	150°C, 1 h	63.6 ± 12.3	0.066	0.914
	180°C, 0.5 h	78.7 ± 0.4	0.062	0.961
	180°C, 1 h	93.7 ± 0.9	0.097	0.937

Pinewood	Untreated	38.7 ± 4.1	0.066	0.973
	150°C, 0.5 h	71.4 ± 3.7	0.094	0.981
	150°C, 1 h	63.3 ± 9.3	0.073	0.933
	180°C, 0.5 h	61.1 ± 4.4	0.080	0.979
	180°C, 1 h	56.0 ± 8.5	0.065	0.962

Rice straw	Untreated	115.9 ± 12.8	0.081	0.943
	150°C, 0.5 h	143.3 ± 7.1	0.084	0.946
	150°C, 1 h	152.7 ± 20.2	0.088	0.918
	180°C, 0.5 h	93.8 ± 19.9	0.078	0.991
	180°C, 1 h	113.4 ± 1.6	0.068	0.984
